# HDL functionality in type 1 diabetes: enhancement of cholesterol efflux capacity in relationship with decreased HDL carbamylation after improvement of glycemic control

**DOI:** 10.1186/s12933-022-01591-9

**Published:** 2022-08-12

**Authors:** Damien Denimal, Serge Monier, Isabelle Simoneau, Laurence Duvillard, Bruno Vergès, Benjamin Bouillet

**Affiliations:** 1 INSERM LNC UMR1231, University of Burgundy, Dijon, France; 2 Department of Biochemistry - Plateforme de Biologie Hospitalo-Universitaire, CHU Dijon, Dijon, France; 3 Department of Endocrinology-Diabetology, CHU Dijon, Dijon, France

**Keywords:** Carbamylation, Cholesterol efflux, HDL, Type 1 diabetes

## Abstract

**Background:**

Reduced cholesterol efflux capacity (CEC) of HDLs is likely to increase cardiovascular risk in type 1 diabetes (T1D). We aimed to assess whether improvement of glycemic control in T1D patients is associated with changes in CEC in relation with changes in carbamylation of HDLs.

**Methods:**

In this open-label trial, 27 uncontrolled T1D patients were given a three-month standard medical intervention to improve glycemic control. HDL fraction was isolated from plasma, and CEC was measured on THP-1 macrophages. Carbamylation of HDLs was evaluated by an immunoassay. Control HDLs from healthy subjects were carbamylated in vitro with potassium cyanate.

**Results:**

HbA_1c_ decreased from 11.4% [10.2–12.9] (median [1st–3rd quartiles]) at baseline to 8.1% [6.6–9.0] after the three-month intervention (P < 0.00001). The CEC of HDLs increased after intervention in 19 (70%) patients (P = 0.038). At the same time, the carbamylation of HDLs decreased in 22 (82%) patients after intervention (P = 0.014). The increase in CEC significantly correlated with the decrease in carbamylated HDLs (r = −0.411, P = 0.034), even after adjustment for the change in HbA_1c_ (β = −0.527, P = 0.003). In vitro carbamylation of control HDLs decreased CEC by 13% (P = 0.041) and 23% (P = 0.021) using 1 and 10 mmol/L of potassium cyanate, respectively.

**Conclusions:**

The improvement of CEC in relation to a decrease in the carbamylation of HDLs may likely contribute to the beneficial cardiovascular effect of glycemic control in T1D patients.

*Trial registration*: NCT02816099 ClinicalTrials.gov.

## Background

Patients with type 1 diabetes (T1D) have an excess risk of death from cardiovascular causes, depending on multiple factors such as age, diabetes duration and nephropathy. The level of glycemic control is also associated with the risk of cardiovascular death in T1D [1]. Long term observational studies (DCCT-EDIC) have shown that interventions to intensify glycemic control have a beneficial effect on macrovascular complications [2, 3].

T1D patients exhibit lipid disorders which likely play a role in their increased cardiovascular risk [4, 5]. Firstly, quantitative lipoprotein abnormalities, such as hypertriglyceridemia or elevated non-HDL cholesterol, are observed in T1D patients with poor glycemic control [6, 7]. However, the lipid profile differs in well-controlled T1D patients, who have normal or slightly decreased serum triglyceride and LDL-cholesterol levels [5, 8].

T1D patients also exhibit qualitative lipoprotein abnormalities that are potentially atherogenic. As far as HDLs are concerned, we and others found a triglyceride enrichment of HDLs from T1D patients. This abnormality seems to be more pronounced in patients with poor glycemic control [9–11], and is attributed to an increased cholesteryl ester transfer between lipoproteins [12, 13]. Moreover, our team previously showed that moderate abnormalities are found in the HDL phosphosphingolipidome of individuals with T1D [9]. Chemical modifications in HDLs, such as oxidation, glycation and carbamylation are of particular interest in T1D. Carbamylation (carbamoylation *stricto *sensu) of HDL proteins is a non-enzymatic irreversible process mediated by isocyanate, and corresponds to the binding of a carbamoyl moiety (–CONH_2_) to lysine, resulting in carbamyl-lysine. Isocyanate originates from either the spontaneous dissociation of urea or from the myeloperoxidase (MPO)-catalyzed oxidation of thiocyanate, or at a lesser extent from tobacco smoke. It is noteworthy that several studies have confirmed that plasma MPO levels are increased in T1D patients [14–16].

Qualitative alterations in the HDLs of T1D patients may result in dysfunctional HDLs, potentially contributing to an increased risk of developing cardiovascular diseases (CVD) [8]. The functional impairment of proteins through carbamylation is of particular relevance seeing as clinical studies have shown that the level of carbamylated proteins are an independent risk factor for CVD [17]. Some studies have found that carbamylation alters the functionality of HDLs [18–21]. For instance, carbamylated HDLs appear to have altered anti-oxidant properties [19], to enhance monocyte to endothelial cell adhesion [20], to activate NF-kB pathway in endothelial cells [20], and to decrease the migration and proliferation of endothelial cells [21]. Interestingly, recent studies have shown that carbamylated HDLs are independently associated with mortality and cardiovascular outcomes in type 2 diabetic patients with relatively well-preserved kidney function [20, 22]. To our knowledge, carbamylation of HDLs has not yet been assessed in T1D, and even less so in the context of an intervention improving glycemic control.

Cholesterol efflux capacity (CEC) is one of the most studied and well-recognized atheroprotective functions of HDLs. In the last few years, CEC has emerged as a better predictor of cardiovascular risk than serum HDL cholesterol levels alone. Studies evaluating CEC in T1D are scarce and the results are still controversial. Two studies reported that CEC was decreased in young patients and in adults with T1D [23, 24]. On the contrary, CEC was found to be enhanced in two other studies carried out on moderately-controlled T1D [13, 25]. To our knowledge, the influence of the improvement of glycemic control on CEC has never been studied in patients with T1D.

In this study, we aimed to explore if improvement of glycemic control in T1D patients is associated with changes in CEC in relation with changes in HDL carbamylation levels.

## Methods

### Study design and participants

This single-center open-label study was carried out at the University Hospital of Dijon, France (Clinical trial reg. no. NCT02816099 ClinicalTrials.gov), and was approved by our regional ethics committee. The recruitments took place from June 2016 to October 2020, and written informed consent was obtained for all patients included in the study. We calculated that a sample size of 22 patients was necessary, considering a two-sided α error of 0.05, a β error of 0.2, an effect size of 0.8 and a standard deviation in CEC changes of 1.25%. We included 27 T1D patients with HbA_1c_ > 75 mmol/mol (> 9%). Blood samples were withdrawn at inclusion and three months after a standard medical intervention to improve glycemic control (i.e. insulin introduction, pump therapy, therapeutic education). All subjects were older than 18 years and were not taking any treatment that could interfere with lipoprotein metabolism (lipid-lowering agents, anti-HIV agents, combined oral contraceptive pill, corticosteroids or retinoic acid). None were pregnant. Microvascular complications (retinopathy, nephropathy) were recorded. Three stages were used to define nephropathy: 0 for no nephropathy, 1 for microalbuminuria between 30 and 300 mg per day and 2 for proteinuria > 300 mg per day.

Blood was collected in the fasting state in BD Vacutainer tubes (Becton Dickinson, Franklin Lakes, USA) in dry tubes and in tubes with EDTA as anticoagulant and preservative. The serum was immediately used after centrifugation for routine lipid measurements. The plasma was immediately separated by centrifugation, and frozen at -80 °C until HDL isolation and subsequent analysis.

### HDL isolation

The HDL fraction (density = 1.063–1.210 g/mL) was isolated from 1.0 mL of plasma by sequential flotation ultracentrifugation at 4 °C using a 50.4 rotor in an Optima L80-XP ultracentrifuge (Beckman Coulter, Brea, CA, USA), as previously reported [26]. Briefly, after ultracentrifugation at 33,000 rpm for 18 h (density adjusted at 1.063 g/mL with potassium bromide), the apolipoprotein (apo) B-containing fraction was withdrawn. Then, after a second ultracentrifugation at 40,600 rpm for 21 h (density = 1.210 g/mL), the HDL fraction was collected in 300 µL. The HDL fraction was extensively dialyzed three times against sterilized 0.01% (wt/vol.) EDTA endotoxin-free phosphate buffered saline (PBS) for 18 h at 4 °C in the dark, and then immediately used for analysis.

### Routine analytical procedures

Total cholesterol, HDL-cholesterol, triglycerides, apolipoprotein AI (apoAI), total proteins, urea and creatinine were measured on a Dimension Vista analyzer using dedicated reagents (Siemens Healthcare Diagnostics, Deerfield, IL, USA). Free cholesterol and phospholipids were measured on the same analyzer but using reagents from Diasys (Condom, France). LDL cholesterolemia was estimated by the Friedewald equation, since triglyceridemia was below 3.88 mmol/L in each subject. Esterified cholesterol in each HDL fraction was calculated as the difference between total and free cholesterol. HbA_1c_ was measured with a G8 HPLC Analyzer (Tosoh Bioscience, Tokyo, Japan). MPO was assessed by a commercial kit (Mercodia, Uppsala, Sweden) in plasma obtained from EDTA tubes. The estimated glomerular filtration rate (eGFR) was calculated with the Chronic Kidney Disease—Epidemiology Collaboration (CKD-EPI) equation.

### Cholesterol efflux capacity (CEC)

The primary end point of this study was the change in CEC after the intervention to improve glycemic control. The capacity of HDLs to support cholesterol efflux was analyzed using the human monocyte cell line THP-1 (Sigma-Aldrich, Saint-Louis, MO, USA). THP-1 cells were maintained in RPMI 1640 complete medium (Gibco, New York, NY, USA) containing 4.5 g/L d-glucose, 2.383 g/L HEPES buffer, L-glutamine, 1.5 g/L sodium bicarbonate, 100 mg/L sodium pyruvate, and supplemented with 10% (vol./vol.) fetal bovine serum (Thermo-Scientific, Illkirch, France) and 1% (vol./vol.) penicillin–streptomycin (Thermo-Scientific). Cells were cultured in exponential phase at day 1. At day 2, cells were seeded for 48 h (37 °C, 5% CO_2_) into 96-well plates at density of 10^5^ cells in 200 µL per well with 100 nmol/L phorbol 12-myristate-13-acetate in complete medium. At day 4, cells were charged for 1 h (37 °C, 5% CO_2_) with 10 nmol/L Bodipy-labelled cholesterol in ethyl acetate (TopFluor^®^, Sigma-Aldrich) in complete medium containing 2 µg/mL Sandoz 58–035 (Sigma-Aldrich) as acyl-CoA:cholesterol acyltransferase inhibitor, 20 mmol/L methyl-β-cyclodextrin (TCI, Paris, France) and 10 µmol/L TO901317 (Sigma-Aldrich) as liver X receptor (LXR) agonist to enhance the expression of ATP-binding cassette (ABC) transporters. After removal of medium, cells were incubated overnight in RPMI 1640 glutamax, 0.2% (vol./vol.) fatty acids free bovine serum albumin, 2 µg/mL Sandoz 58–035, 10 µmol/L TO901317. At day 5, HDL fractions (20 µg/mL apoAI) were incubated for 4 h (37 °C, 5% CO_2_) in 1X Opti-Klear™ live cell imaging buffer (Abcam, Cambridge, UK) containing 2 µg/mL Sandoz 58–035 and 10 µmol/L TO901317. After 4 h, 80 µL of supernatant were transferred into a black 96-well plate and fluorescence was read at 485/535 nm using a SPARK microplate reader platform (Tecan, Grödig, Austria). The remaining medium was withdrawn and cells were lysed by adding 100 µL/well of reporter lysis buffer (Promega, Madison, WI, USA). Eighty microliters of lysate were transferred into a black 96-well plate and fluorescence was read in the same conditions as the supernatant. Each fluorescence value in supernatant and in lysate was corrected by autofluorescence obtained in wells containing HDLs but without Bodipy cholesterol. Percent efflux was calculated by the following formula: [fluorescence in supernatant ÷ (fluorescence in supernatant + fluorescence in lysat)] × 100. All assays were performed in triplicate. All of the samples from one T1D patient (at baseline and post-intervention) were treated in the same runs to avoid bias due to inter-assay variations. To correct for inter-assay variations across plates, a plasma control from a healthy volunteer was included on each plate, and values for plasma samples from patients were normalized to this value in subsequent analyses.

### Carbamylated HDL concentration

Carbamylated proteins were measured in isolated HDLs by a commercial sandwich ELISA (OxiSelect™, Cell Biolabs, San Diego, CA, USA) detecting carbamyl-lysine residues. Each sample was assayed in duplicate. The results were expressed as ng HDL carbamylated proteins / mg HDL total proteins.

### In vitro carbamylation of HDLs

A pool of dialyzed HDLs isolated from healthy subjects (4 mg protein/mL) was incubated with 1 mmol/L and 10 mmol/L potassium cyanate (KCN) in PBS containing 100 μmol/L diethylenetriaminepentaacetic acid (Sigma-Aldrich) for 4 h at 37 °C, as previously described [27]. Control HDLs were incubated under the same conditions with PBS instead of potassium cyanate. HDLs were then immediately and extensively dialyzed three times against sterilized 0.01% (wt/vol.) EDTA endotoxin-free PBS for 18 h at 4 °C in the dark, and then immediately used for analysis.

### Statistics

Data are reported as median [1st–3rd quartiles], otherwise indicated. Statistical calculations were performed using XLSTAT (version 2021.4.1). The primary end point was the change in CEC after the 3-month intervention. Skewness of each continuous variable was assessed using Pearson's first skewness coefficient, and values were log10 transformed before any statistical analysis to improve normality if necessary. The results from patients with T1D before and after the intervention were compared using the non-parametric Wilcoxon signed-rank test for paired samples. Univariate Spearman correlation coefficients were calculated to explore relationships between the changes in CEC after the intervention, and clinical and biological variables. A two-tailed probability level of 0.05 was considered as statistically significant.

## Results

### Clinical and biological characteristics

The clinical and biological characteristics of the 27 T1D patients at baseline and after 3 months of intervention are shown in Table 1. Glycemic control, which was poor at inclusion (HbA_1c_ = 11.4% [10.2–12.9]), decreased by 3.3 units after the 3-month intervention (P < 0.00001). The body mass index (BMI) increased by 2.0 units after the intervention, which is common after a significant improvement in the glycemic control of poorly controlled T1D patients. Plasma urea levels were similar at baseline and after intervention (P = 0.82). Serum HDL-cholesterol increased in 20 (74%) subjects (P = 0.002).

**Table 1:** Clinical and biological characteristics of patients with type 1 diabetes

	At baseline (n = 27)	After intervention (n = 27)	P-value
Demographic and clinical characteristics			
Age (years)	30.8 [26.2–33.1]	31.1 [26.4–33.3]	
Sex ratio (M/F)	15 / 12		
Body mass index (kg/m^2^)	20.3 [19.0–22.8]	22.3 [20.6–24.7]	0.0003
Tobacco users, n (%)	13 (48)		
Retinopathy, n (%)	4 (15)		
Nephropathy, n (%)			
Stage 0	26 (96)		
Stage 1	0 (0)	13 (48)	1.0
Biological characteristics			
HbA_1c_ (mmol/mol)	101 [88–118]	65 [49–75]	< 0.00001
HbA_1c_ (%)	11.4 [10.2–12.9]	8.1 [6.6–9.0]	< 0.00001
eGFR (ml.min^−1^.1.73 m^−2^)	126 [119–132]	124 [116–128]	0.06
Plasma urea (mmol/L)	5.10 [4.40–6.60]	5.20 [4.35–5.95]	0.82
Serum triglycerides (mmol/L)	0.94 [0.86–1.57]	0.95 [0.82–1.15]	0.10
Serum total cholesterol (mmol/L)	4.50 [3.80–5.12]	4.40 [4.00–4.70]	0.54
Serum HDL-cholesterol (mmol/L)	1.27 [1.08–1.51]	1.44 [1.23–1.76]	0.002
Serum LDL-cholesterol (mmol/L)	2.55 [2.09–3.04]	2.40 [1.87–2.91]	0.12
Plasma MPO (µg/L)	85.5 [68.8–104.3]	78.9 [60.3–104.3]	0.51
HDL composition			
HDL-proteins (% HDL total weight)	43.4 [42.0–44.4]	43.5 [42.2–44.8]	0.73
HDL-apoAI (% HDL total weight)	31.3 [29.7–31.9]	31.0 [29.9–31.8]	0.60
HDL-phospholipids (% HDL total weight)	32.9 [32.3–35.1]	33.6 [32.4–34.6]	0.35
HDL-esterified cholesterol (% HDL total weight)	18.3 [17.1–20.0]	18.7 [17.4–20.2]	0.23
HDL-triglycerides (% HDL total weight)	4.23 [3.53–4.77]	3.59 [2.74–4.19]	0.011
HDL-esterified cholesterol/HDL-triglycerides	8.24 [5.94–9.44]	9.28 [7.55–13.34]	0.005
HDL-free cholesterol (% HDL total weight)	3.42 [2.99–3.84]	3.42 [3.22–3.99]	0.051

Data are medians [1st and 3rd quartile], otherwise indicated. P-values were obtained using the non-parametric Wilcoxon signed-rank test for paired samples

eGFR, estimated glomerular filtration rate; MPO, myeloperoxidase

The composition of HDLs at baseline and after the 3-month intervention is also shown in Table 1. The content of HDL fractions in proteins, phospholipids, apoAI and esterified cholesterol did not change after the intervention. HDLs were 15% poorer in triglycerides after intervention (P = 0.011). At the same time, the ratio of esterified cholesterol/triglycerides in HDLs significantly increased by 13% (P = 0.005). The content of HDLs in free cholesterol tended to be higher after the intervention (P = 0.051).

### Cholesterol efflux capacity (CEC)

**Fig. 1:**
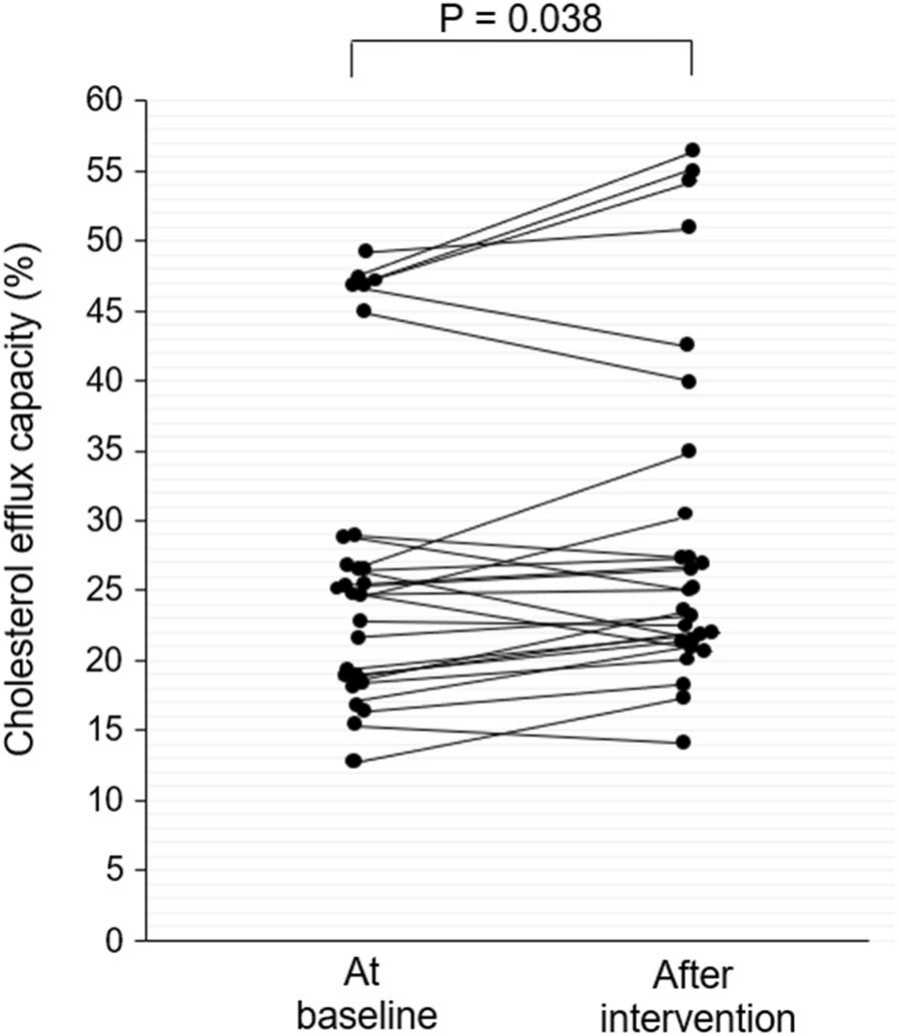
Changes in cholesterol efflux capacity after intervention

The medians [1st and 3rd quartile] of CEC were 25.3% [18.5–29.0] at baseline and 25.3% [21.4–33.0] after the 3-month intervention. Interestingly, the CEC of HDLs significantly increased after the intervention in 19 (70%) T1D patients (P = 0.038) (Fig. 1).

### Carbamylation of HDLs

**Fig. 2:**
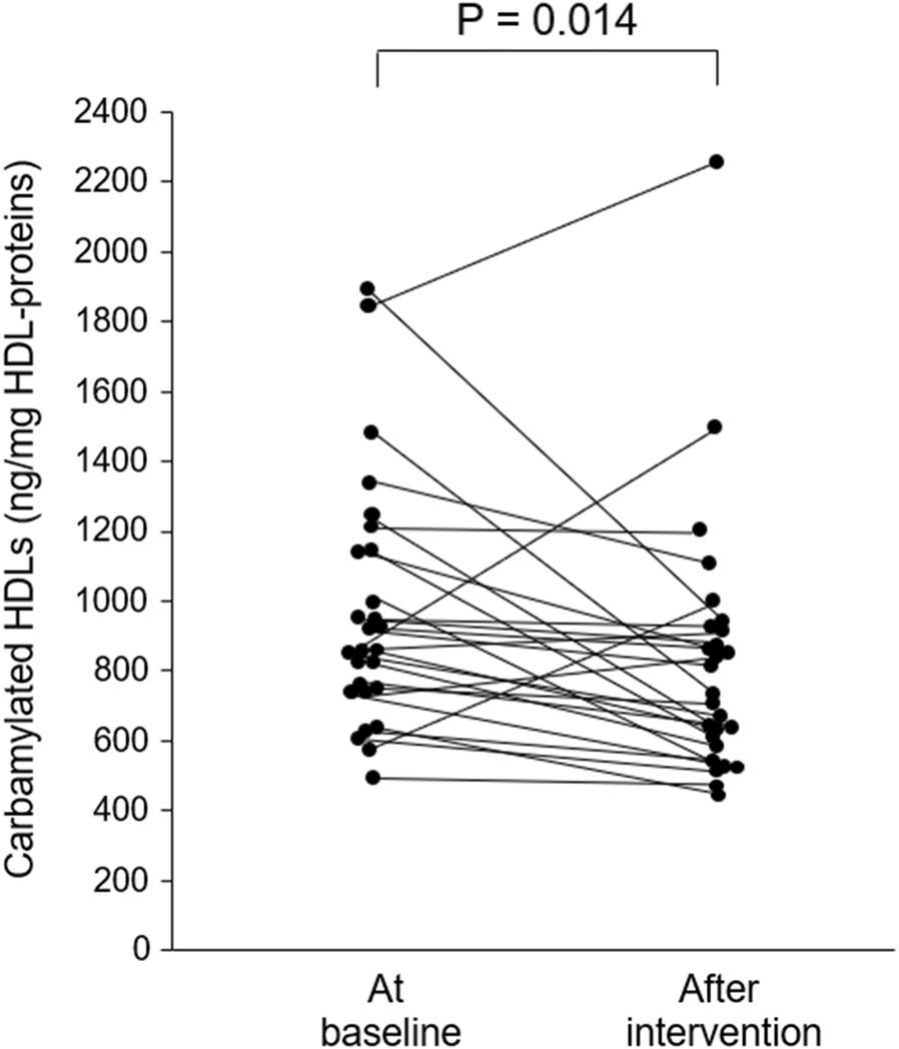
Changes in carbamylated HDL levels after intervention

The content of carbamylated proteins in HDL fraction significantly decreased in 22 (82%) T1D patients after the intervention (from 880 [758–1151] to 728 [620–942] ng/mg HDL-proteins, P = 0.014) (Fig. 2).

### Correlations

**Fig. 3:**
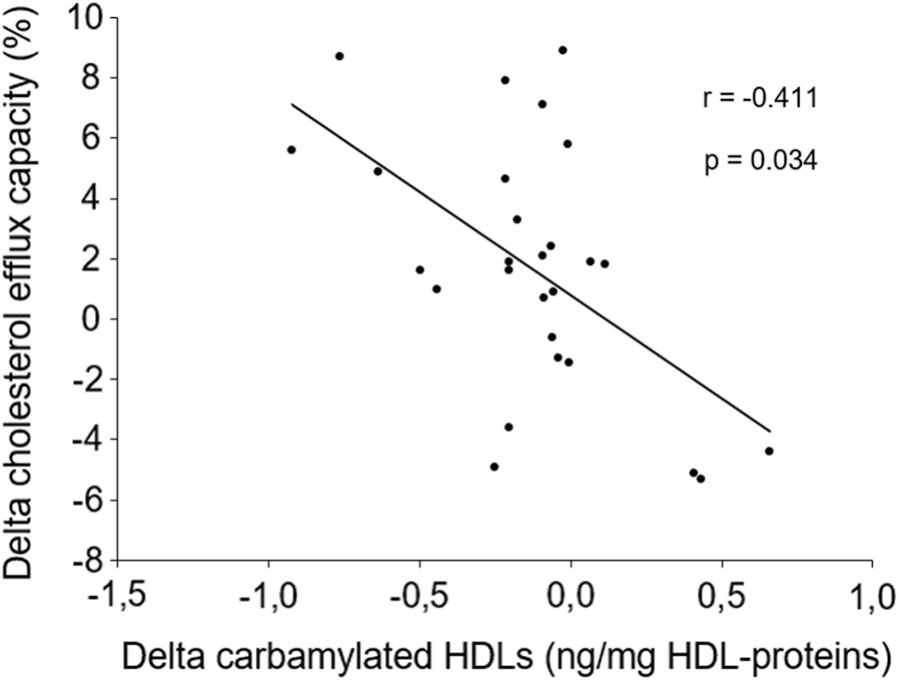
Correlation between changes in cholesterol efflux capacity and in carbamylated HDL levels after intervention

The univariate correlation coefficients between changes in CEC and other clinical and biological parameters are presented in Table 2. The enhancement of CEC after the 3-month intervention significantly correlated with the decrease in carbamylated proteins in HDLs (r = −0.411, P = 0.034) (Fig. 3) and with the change in HbA_1c_ (r = −0.403; P = 0.038). The absolute levels of CEC and carbamylated HDLs were not associated neither at baseline (r = −0.116, P = 0.56) nor after intervention (r = −0.283, P = 0.15). The absolute levels of CEC and HbA_1c_ were not associated at baseline (r = −0.186, P = 0.35) and after intervention (r = −0.118, P = 0.56).

**Table 2:** Univariate correlation study

Changes after intervention in:	CEC		Carbamylated HDLs	
	r	P-value	r	P-value
CEC	1	N.A	−0.411	0.034
Carbamylated HDLs	−0.411	0.034	1	N.A
BMI	0.105	0.60	−0.087	0.67
HbA_1c_	−0.403	0.038	0.141	0.48
Serum triglycerides	−0.068	0.73	0.109	0.59
Serum HDL-cholesterol	0.193	0.33	−0.494	0.010
MPO	−0.005	0.98	0.153	0.44
eGFR	−0.080	0.69	−0.094	0.23
Urea	0.109	0.59	0.158	0.32
HDL-phospholipids	0.189	0.34	0.173	0.39
HDL-proteins	0.059	0.77	−0.446	0.021
HDL-triglycerides	0.123	0.54	−0.516	0.006
HDL-esterified cholesterol	−0.169	0.40	0.032	0.87
HDL-free cholesterol	0.147	0.46	−0.492	0.010
HDL-apoAI	−0.140	0.48	−0.080	0.69

r corresponds to the Spearman correlation coefficient. *BMI* body mass index, *CEC* cholesterol efflux capacity, *eGFR* estimated glomerular filtration rate, *MPO* myeloperoxidase, *N.A.* not applicable.

After multivariate analysis, the enhancement of CEC after intervention remained significantly associated with carbamylated HDLs (β = −0.527, 95% confidence interval (CI) [−0.858 to −0.195], P = 0.003), but not with HbA_1c_ (β = −0.283, 95% CI [−0.615–0.049], P = 0.09). Interestingly, the relationship between changes in CEC and in carbamylated HDLs remained significant after adjustment for smoking status (β = −0.541, 95% CI [−0.886 to −0.195], P = 0.004). Moreover, it is noteworthy that the increase in CEC after the intervention did not correlate with other HDL metrics.

### In vitro carbamylation of HDLs

**Fig. 4:**
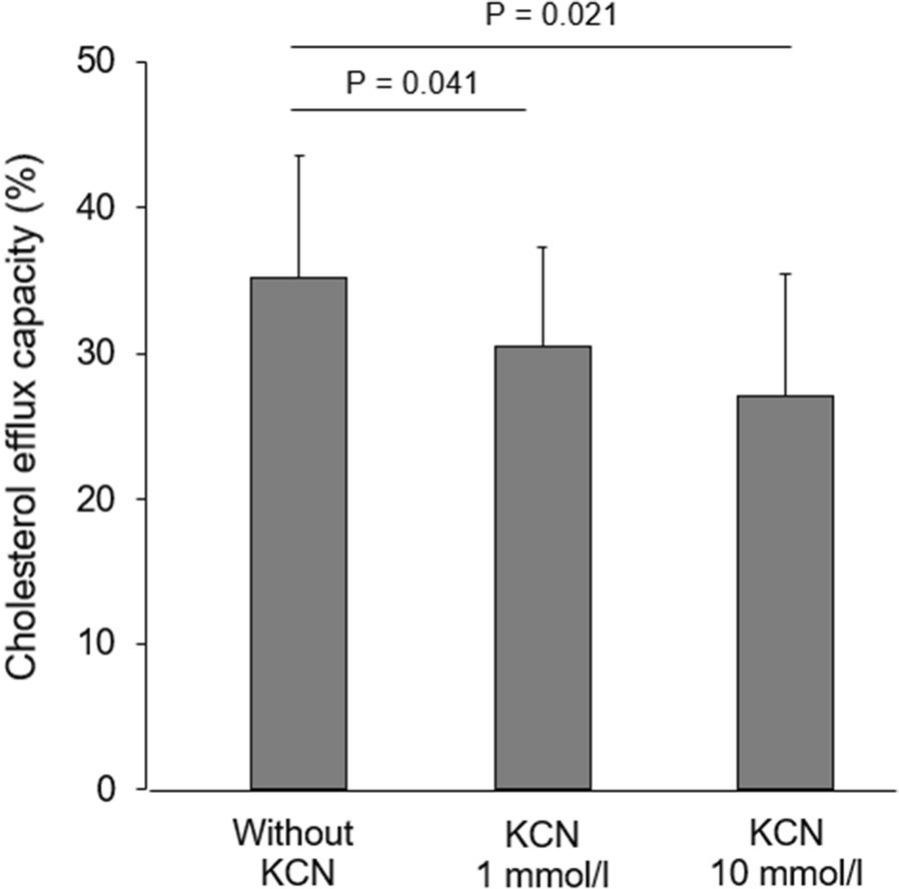
Effect of in vitro HDL carbamylation on cholesterol efflux capacity. Data are presented as means ± SD. Data are obtained from 9 replicates obtained in three independent experiments. KCN: potassium cyanate

In vitro carbamylation of HDLs isolated from healthy subjects was associated with significant decreases in CEC of 13% (P = 0.041) and 23% (P = 0.021) using 1 and 10 mmol/L of potassium cyanate, respectively (Fig. 4). Carbamylated proteins in HDL fraction treated with PBS, 1 and 10 mmol/L potassium cyanate were 350, 652 and 8 400 ng/mg HDL-proteins, respectively.

## Discussion

This study is the first to investigate changes in CEC in combination with HDL carbamylation levels in T1D patients after an intervention to improve glycemic control. After the three months of intervention, the median of HbA_1c_ (expressed in %) considerably reduced by 3.3 units. At the same time, we found that CEC significantly rose after the intervention for glycemic control. This result is of particular interest considering the emerging evidences linking CEC and CVD [28]. For instance, in a recent prospective study, a one SD increase in CEC at baseline was associated with a 27% drop in incident CVD [29].

There is little published data connecting CEC and glycemic control levels in T1D. A previous study found no difference in CEC between 25 T1D adolescents with fair glycemic control (HbA_1c_ 57 ± 10 mmol/mol, 7.3 ± 1.2%) and 27 T1D adolescents with poor glycemic control (HbA_1c_ 103 ± 21 mmol/mol, 11.6 ± 2.16%) [11]. However, it was a case–control study design, contrary to our interventional study. CEC was also evaluated in Fu5AH rat hepatoma cells in this previous study, compared to human THP-1 cells in our present study. Fu5AH cells exhibit a high level of SR-BI expression but lack functional ABCA1, a major transporter involved in cholesterol efflux from human macrophages [30]. Therefore, this cellular model may be less relevant and less sensitive than human THP-1 cells. In another case–control study, Manjuhata et al. also found no difference in CEC between 15 poorly-controlled (HbA_1c_ 86 ± 11 mmol/mol, 10.0 ± 1.22%) and 15 well-controlled (HbA_1c_ 45 ± 3 mmol/mol, 6.3 ± 0.49%) T1D adults, using J774 murine cells [24]. However, the relatively low number of participants could be the reason for the difference with our interventional study. Unfortunately, the two other studies evaluating CEC in T1D did not provide data depending on glycemic control levels, making any comparison impossible [13, 23]. One of the strengths of our study is that we evaluated the influence of glycemic control longitudinally in the same subjects.

The HDL content in total phospholipids and apoAI are key determinants of CEC [31–33]. However, we found no difference in these metrics after intervention (Table 1), making it unlikely that these factors played a role in the CEC variations in our cohort. Although the HDL contents in total proteins and apoAI were not modified, we cannot definitely exclude a change after intervention in the distribution between HDLs containing only apoAI (LpAI) and those containing both apoAI and apoAII (LpAI:AII). ApoAII is the second most abundant scaffold protein in HDLs, and emerging evidences support functional properties of apoAII in LpAI:AII. Indeed, the presence of apoAII in HDLs isolated from human or transgenic rabbits enhanced CEC compared to HDL containing only apoAI [34, 35]. Thus, we cannot exclude that a potential enrichment in LpAI:AII after intervention in our cohort could contribute to the improvement in CEC.

The triglyceride content in HDLs decreased after the intervention, and there was a significant increase in the esterified cholesterol/triglycerides ratio. The triglyceride content of HDLs modulates the exposure of apoAI to the aqueous phase [36], with functional consequences such as altered antioxidant properties [37]. Thus, an enrichment in triglycerides may impair the binding of HDLs to the transporters on macrophages, leading to a decrease in CEC. Subsequently, we can hypothesize that the decrease in triglycerides in HDLs after intervention could have beneficial effects on CEC. However, we found no correlation between the increase in CEC after intervention and changes in HDL content in triglycerides.

Furthermore, we cannot not exclude that a decrease in HDL glycation after intervention may play a role in improving the CEC, even if we were unable to assess the HDL glycation levels in our cohort unfortunately. There are conflicting results about the effect of HDL glycation on CEC. It has been shown that in vitro glycation impaired HDL-mediated CEC, which was restored by addition of advanced glycation end products inhibitors [38]. But, this has not been observed in a previous paper [39]. In humans, the CEC negatively correlated with HbA_1c_ level in type 2 diabetes [40]. It is noteworthy that in our cohort of patients with type 1 diabetes, the changes in CEC and in HbA_1c_ after intervention were associated in univariate analysis. Although this is no longer true after multivariate analysis, it remains plausible that a decrease in HDL glycation after intervention contributes at least in part to the improvement of the CEC.

An important result in our study was the decrease in the carbamylation of HDLs following the 3-month intervention. To our knowledge, this is the first study to provide data about the carbamylation of HDLs in T1D. There are some published reports in type 2 diabetes, but without comparison in carbamylated HDL levels between different glycemic control subgroups [20, 22]. Spontaneous dissociation of urea is a well-known source of isocyanate and therefore of carbamylation. However, we observed no change in the urea plasma levels after the intervention improving glycemic control, suggesting that change in urea level after intervention is likely not a cause of the decrease in carbamylated HDLs in our study. Moreover, since MPO is also a major source of carbamylation, we evaluated MPO levels in our cohort, and we observed no decrease after the intervention. Several studies reported elevated MPO levels in T1D [14–16], but, to our knowledge, there is no previous study evaluating MPO levels according to glycemic control in T1D. However, our results may be in line with a recent case–control study showing no correlation between MPO and HbA_1c_ or fasting glucose levels in patients with type 2 diabetes [20]. Another source of exposure to isocyanate is tobacco smoke [41], and plasma levels of thiocyanate, the precursor of cyanate, is increased in smokers [42]. However, none of the smokers in our cohort stopped smoking during the intervention. Moreover, the relationship between changes in CEC and carbamylated HDL remained significant after adjustment for smoking status. So, it is unlikely that changes in smoking status are responsible for the decrease in the carbamylation of HDLs. All these considerations taken together, the mechanisms underlying the decrease in carbamylation observed in our cohort after intervention remain elusive.

Interestingly, we found that the post-intervention increase in CEC was strongly correlated with the decrease in carbamylated proteins in HDLs. This observation prompted us to assess the impact of in vitro carbamylation of HDLs on CEC in our cell model. We found that in vitro carbamylation of healthy HDLs altered CEC. Previous data have shown that the carbamylation of HDLs significantly reduced their ability to promote cholesterol efflux in SR-BI-overexpressing THP-1 cells [27]. These authors observed no altered cholesterol efflux with carbamylated HDLs using unmodified THP-1 cells [27]. Unlike us, they did not use a LXR agonist to induce the expression of ABC transporters, which could be an explanation for this discrepancy. While the mechanisms behind the decreased CEC of carbamylated HDLs are not fully understood, it has been demonstrated that the carbamylation of HDLs increases binding affinity to SR-BI and leads to an accumulation of cholesterol in macrophages [27]. Moreover, lecithin-cholesterol acyltransferase (LCAT), which is involved in the maturation of spherical HDLs and in the initial step of reverse cholesterol transport, has been shown to be less active with carbamylated HDLs [19]. Finally, we could not exclude that the carbamylation of apoAI decreases its half-life in circulation, as shown for the glycation of apoAI [42]. Thus, the decrease in carbamylated HDLs after intervention could contribute to the changes in HDL-cholesterol levels and in cholesterol efflux. However, it is unlikely that such a mechanism explains our results because our CEC assay was adjusted for apoAI levels.

The present study has limitations and potential reasons for caution. First, we used only samples from a single center. Secondly, we have no measurement of glycation levels of HDLs in our cohort. Another potential limitation of our work is the fact that we did not perform CEC determinations using murine J774 cells, which is the main model used in clinical studies reporting the impact of CEC on incident CVD events in general population cohorts. However, using human cells such as THP-1 seems at least as relevant as murine J774 cells, and a recent study using THP-1 cells also demonstrated a link between CEC and CVD [29].

## Conclusion

This study shows for the first time that the improvement of glycemic control in patients with T1D is associated with an increase in CEC in relation with a decrease in the carbamylation of HDLs. Further investigations are needed to understand the mechanisms behind the decrease in HDL carbamylation following improvement of glycemic control.

## Data Availability

The datasets used and/or analysed during the current study are available from the corresponding author on reasonable request.

## Abbreviations

ABC: ATP-binding cassette
CEC: Cholesterol efflux capacity
CKD-EPI: Chronic Kidney Disease—Epidemiology Collaboration
EDIC: Epidemiology of Diabetes Interventions and Complications
LXR: Liver-X-receptor
MPO: Myeloperoxidase
